# M for measles, M for math, M for mod**...**

**DOI:** 10.3325/cmj.2019.60.463

**Published:** 2019-10

**Authors:** Branimir K. Hackenberger

**Affiliations:** Department of Biology, Josip Juraj Strossmayer University, Osijek, Croatia *hackenberger@biologija.unios.hr*

What is common to mathematics and measles? Except for the letter M, and except that at least someone likes mathematics (although this, in a statistical language, is a rare event), there are no similarities. However, mathematics deals with many things, perhaps with everything imaginable, including measles. Specifically, infectious diseases, just like all the things we fear, are always an exciting research topic. Not only for epidemiologists and physicians but for other experts and scientists, including mathematicians. Also, mathematics and statistics offer solutions to many problems related to the spread of infectious diseases (although these solutions are, at least initially, only theoretical).

There is one big problem with infectious diseases today, especially in the developed Western world. Namely, contagious diseases are no longer among the first causes of mortality. Education, better hygiene, and, above all, vaccination, together with the development of public health, have pushed the mortality from infectious diseases far below the mortality from cardiovascular disease and cancer. According to the World Health Organization, in some countries, there is no longer mortality from infectious diseases, except from influenza, on an annual basis. Ebola virus disease and other infectious diseases with high mortality are expected only in poor underdeveloped countries. In the West, the risk of infection and death from a contagious disease is very small. Therefore, our fear has also been reduced proportionally. It has been reduced to such an extent that hygiene habits are worsening and health protection has been reduced to vaccination only.

Moreover, the absence of infectious disease mortality has had the ultimate consequence of providing an impetus for resistance to vaccination. Paradoxically, the so-called anti-vaxers justify their resistance to mandatory vaccination by the absence of epidemics and mortality from infectious diseases, ignoring the fact that infectious diseases in particular areas have been eradicated precisely through vaccination.

Recent outbreaks in various countries have been blamed, among other reasons, on the rise of the anti-vaccination movement, which has spread across social media, discouraging parents from immunizing their children against measles. In 2019, as many as four European countries lost their status of measles-free countries: Albania, the Czech Republic, Greece, and the United Kingdom. In the first half of this year, over 13 000 measles cases were registered in the EU (1). According to the Netherlands National Institute of Public Health and the Environment, by mid-2019 a considerable epidemic had erupted in some European countries: Ukraine, Romania, Poland, the Czech Republic, Slovakia, Italy, France, Bulgaria, Lithuania, and the United Kingdom. Furthermore, the European Center for Disease Prevention and Control reports that, by the release of this *CMJ* issue, no European country will have been free of measles infections (Table 1).

**Table 1:** Measles cases in the European Union during the first half of 2019

Country	Total number of cases
France	2675
Italy	1847
Poland	1582
Romania	1445
Bulgaria	1158
Lithuania	851
United Kingdom	772
Czech Republic	631
Germany	538
Slovakia	448
Belgium	440
Spain	253
Austria	154
Ireland	69
Netherlands	69
Portugal	50
Greece	35
Malta	31
Sweden	27
Estonia	26
Croatia	25
Luxembourg	25
Hungary	24
Slovenia	20
Denmark	18
Norway	17
Finland	16
Iceland	8
Cyprus	6
Latvia	4

This situation has several significant consequences. First, the spread of this infectious disease creates the need for the development of better predictive epidemiological models. Furthermore, the measurable decline in population vaccination in individual countries allows for precise quantification of the impact of vaccination on the spread parameters of this disease. In other words, anti-vaxers have unwittingly contributed to the knowledge of measles epidemiology. Also, such rapid changes in measles prevalence, especially in developed countries, raise many questions not only about measles but also about other infectious diseases, some of which are much more dangerous and deadly. In the time of past great epidemics, the frequency of travel and potential transmission was minimal compared with the situation today. Therefore, the spread of infectious diseases is also much faster. This is why some countries, such as China, have body temperature sensors installed at border crossings (especially at all international airports). Without much hesitation, they quarantine all the people who try to enter the country with a fever – anything to prevent the development of an epidemic.

Although measles is not generally considered a disease with high mortality, in 2017 it killed more than 100 000 people worldwide. In Congo, the measles epidemic that has lasted from 2011 till now has killed over 15 000 people, mostly children. In 2019 alone, 3200 people in Congo have died from measles, more than from Ebola virus (2). This sounds paradoxical given that the mortality rate from Ebola virus is 60% and from measles “only” 2%. However, this information points to other features of measles epidemiology that further add importance to preventing its spread but also indicates the need for computational models and epidemics simulations. In the early 1970s, several interesting scientific articles compared mathematical models for various infectious diseases. In this way, the individual models complemented each other with real data. Although Daniel Bernoulli first used these mathematical models to describe, characterize, and study epidemics, Sir Ronald Ross is considered the first mathematical epidemiologist. In his work at the beginning of the last century, he used differential equations to describe a range of epidemiological phenomena, from vector transmission of malaria and transmission of sexually transmitted diseases to socio-epidemiological conditions. The real application of epidemiological models in practice followed later. In 1978, Longini et al used a model to decide which age and social groups should be the first to be vaccinated against influenza to minimize the costs and mortality. Ten years later, a mathematical model was used to determine the optimal age for measles vaccination. These were serious and successful attempts to apply mathematical models in epidemiological practice. Today, it is almost unthinkable to make decisions without first accurately assessing their consequences. And this is not possible without simulations, computer experiments, expert models, and artificial intelligence-based prediction systems. Therefore, in addition to physicians and other medical staff, mathematicians, statisticians, and programmers also significantly contribute to the fight against measles and other infectious diseases.

**Figure 1:**
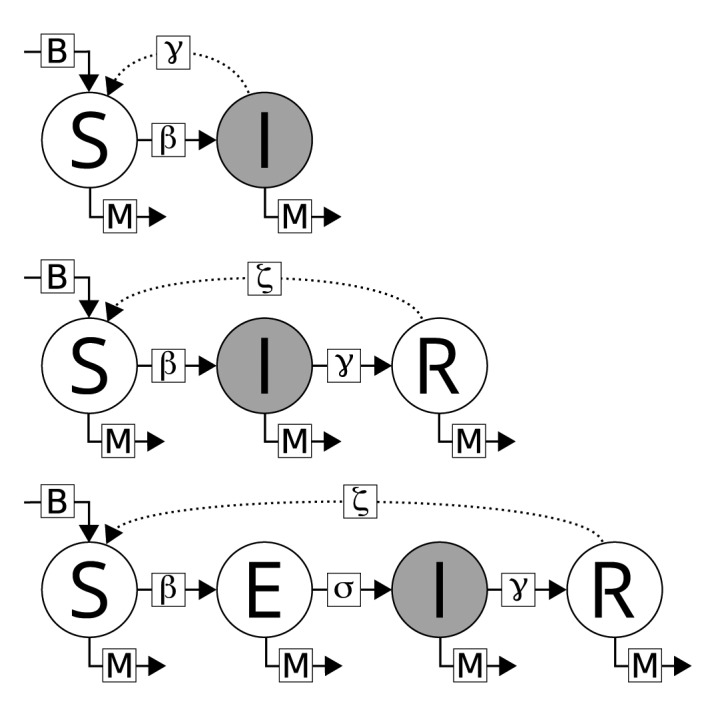
Fundamental epidemic compartmental models.

The most common epidemiological models are the so-called compartmental models. The basis of these models is the assumption that the entire population is divided into groups, ie, compartments. The simplest such model is the SI/SIS model (Figure 1). In this model, the entire population is divided into two compartments: a compartment of susceptible organisms, ie, organisms that can be infected but are not infected, and a compartment of infected or infectious organisms. When in the first compartment an organism becomes infected, it becomes contagious and remains so for the rest of its life (SI) or ceases to be infectious and becomes susceptible to infection (SIS) again. A slightly more complex model is SIR/SIRS. This model consists of three compartments: a compartment with susceptible organisms, a compartment with infected organisms, and a compartment with recovered organisms. If recovered organisms acquire permanent immunity after recovery, they remain in the compartment of recovered organisms (SIR). If the immunity of the recovered organisms is short-term or absent, these individuals are transferred back to the compartment of susceptible organisms (SIRS). SEIR/SEIRS models have an additional compartment that comprises organisms that are infected but are not infectious, ie, undergo the incubation phase. A mathematical description of the three basic epidemiological models is given in Table 2. Among the basic epidemiological models, some authors also include the models with “maternal” compartment (M). Namely, for many infectious diseases, including measles, newborns are protected from the infection by antibodies inherited from the mother. These models are then called MSIR, MSIRS, MSEIR, MSEIRS, etc. Moreover, basic models can also include models that contain a compartment of carrier organisms (C).

**Table 2:** Mathematical description of fundamental compartmental epidemiological models

Model	Example	Differential equations description
SI	Herpes	Without vital dynamics 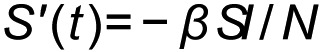 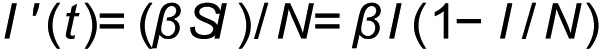 With vital dynamics 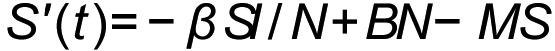 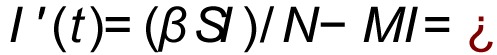
SIS	Gonorrhea	Without vital dynamics 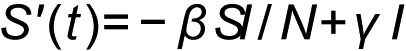 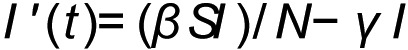 With vital dynamics 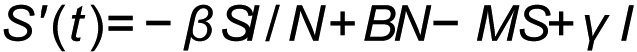 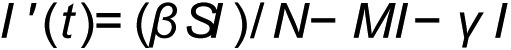
SIR	Measles, mumps	Without vital dynamics 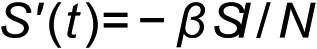 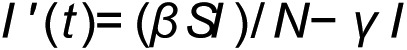 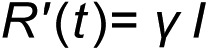 With vital dynamics 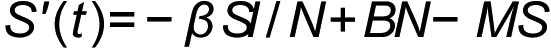 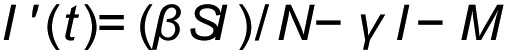 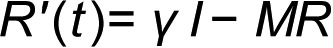
SIRS	Influenza	Without vital dynamics 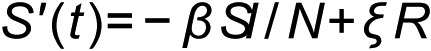 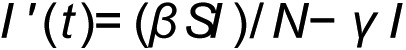 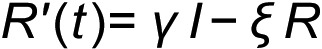 With vital dynamics 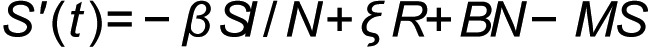 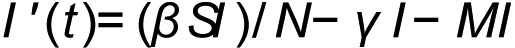 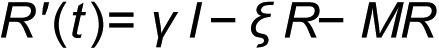
SEIR	Measles, pox, dengue	Without vital dynamics 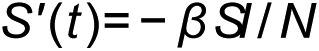 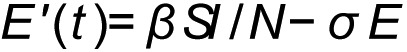 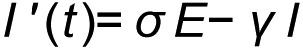 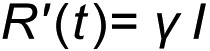 With vital dynamics 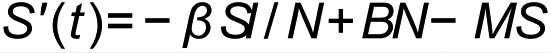 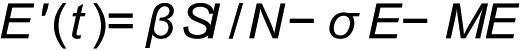 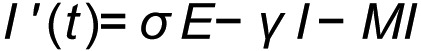 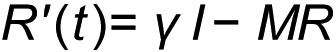

**Figure 2:**
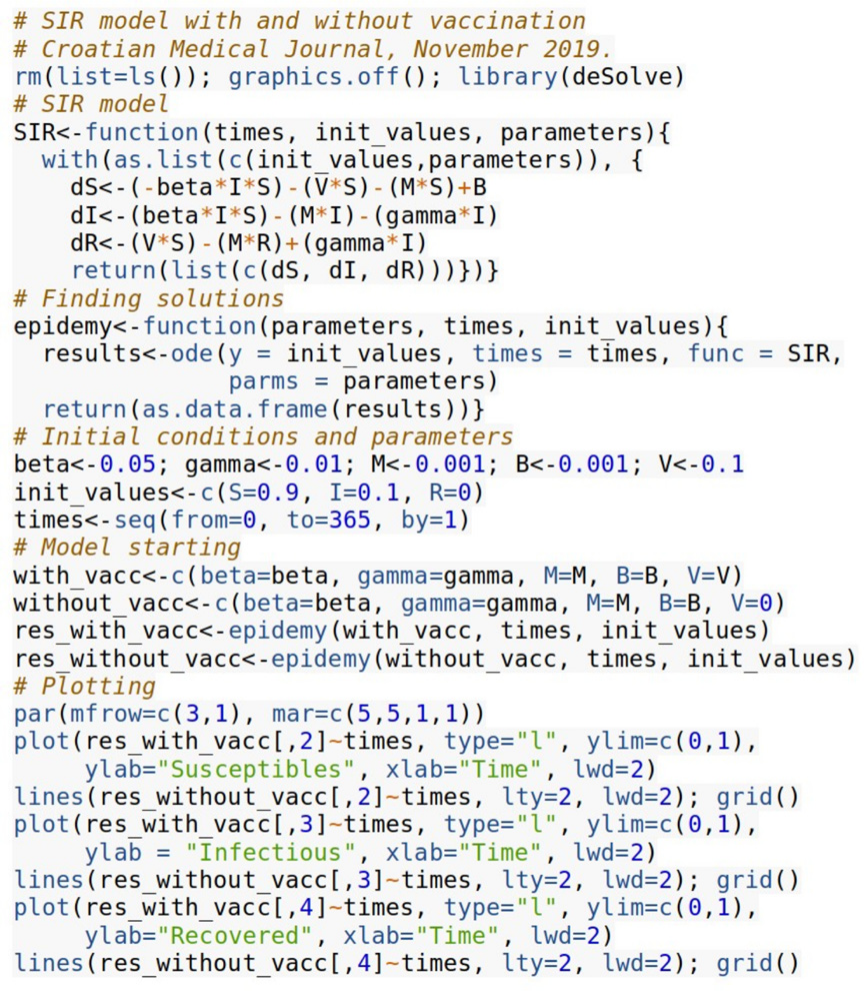
An example of an R-script with a vital dynamics of an SIR model that describes the abundance in all three compartments (Suspicious, Infectious, and Recovered).

**Figure 3:**
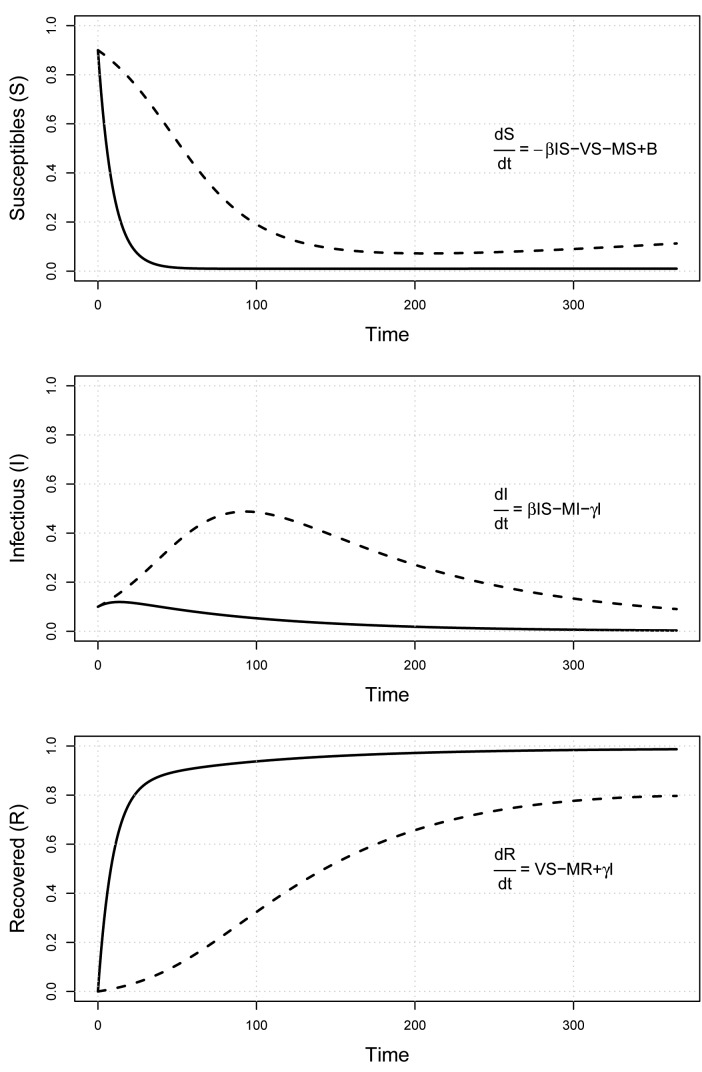
A graphical output of an R-script with a vital dynamics of an SIR model.

Heslop et al (3) gave an excellent overview of free software tools to be used during epidemics. In addition to the ready-made software solutions, there are various tools in the framework of standard computer languages (C ++, Python) or computer environments (R). Table 3 lists the most well-known packages for Python, C ++, and R. Basic models can be set up in any programming language for which there are ready packages with functions for solving differential equations. Figure 2 shows an example of an R-script with a vital dynamics of an SIR model that describes the abundance in all three compartments (Suspicious, Infectious, and Recovered), either in vaccinated and non-vaccinated models, and Figure 3 shows an example of a graphical output of this script. In addition to the basic packages, in this script deSolve package was used to solve differential equations. This figure shows that even a relatively low vaccination rate significantly reduces the number of patients. Such a model (SIR) with discrete age groups was used by Zhou et al (4) to study the transmission dynamics of infectious disease in a host population. Verguet et al (5) have shown that the SIR model of the measles epidemic, despite all its limitations, is an important tool for vaccination decision-making, complementing the existing information from surveillance systems. Erhardt et al (6) made an excellent study using SIR-based mathematical modeling of measles with vaccination and waning immunity, while Dei et al (7), using the SEIR model, investigated the effect of backward bifurcation in controlling measles transmission by vaccination.

**Table 3:** Software packages with functions for epidemiological statistics and modeling

Package	Language	Version	Year of last release	Description
EpiFire	C++		2012	Modeling of the spread of an infectious disease in a population and generation and manipulation of networks of nodes and edges (11).
epipy	Python	0.0.2.1.	2014	A set of tools for analyzing and visualizing epidemiology data. It can currently produce stratified summary statistics, case tree and checkerboard plots, epicurves, analysis of case attribute (eg, sex) by generation, 2 × 2 tables with odds ratio and relative risk, summary of cluster basic reproduction numbers.
epiweeks	Python	2.1.1	2019	A Python package to calculate epidemiological weeks using the CDC (MMWR) and ISO week numbering systems.
dismod-mr	Python	1.1.0	2019	An integrative metaregression framework for descriptive epidemiology.
zEpid	Python	0.8.1	2019	An epidemiology analysis package, providing easy to use tools for epidemiologists coding in Python 3.5+, providing a toolset including basic epidemiology calculations, functional form assessment plots creation, creation of effect measure plots, and causal inference tools.
epi	R	2.38	2019	Functions for demographic and epidemiological analysis in the Lexis diagram, ie, register and cohort follow-up data, including interval censored data and representation of multistate data. Also some useful functions for tabulation and plotting. Contains some epidemiological data sets (12,13).
epibasix	R	1.5.1.	2018	Elementary tools for analysis of common epidemiological problems, ranging from sample size estimation, through 2 × 2 contingency table analysis and basic measures of agreement (kappa, sensitivity/specificity).
epicalc	R	2.15.1.0.	2012	Functions making R easy for epidemiological calculation (14,15).
epiDisplay	R	3.5.0.1.	2018	Functions for epidemiological data exploration and result presentation.
epiR	R	1.0.4.1.	2019	Functions for directly and indirectly adjusting measures of disease frequency, quantifying measures of association on the basis of single or multiple strata of count data presented in a contingency table, and computing confidence intervals around incidence risk and incidence rate estimates.
epitools	R	0.5.10.2.	2017	Numerical tools and programming solutions that have been used and tested in real-world epidemiologic applications.
surveillance	R	1.17.1	2019	Implementation of statistical methods for the modeling and change-point detection in time series of counts, proportions and categorical data, as well as for the modeling of continuous-time epidemic phenomena, eg, discrete-space setups such as the spatially enriched Susceptible-Exposed-Infectious-Recovered (SEIR) models, or continuous-space point process data, such as the occurrence of infectious diseases (16).

These models are decades old. They served as a basis for a whole range of very complex epidemiological models. But the real “trick” of modern computer epidemiology is Bayesian statistics. Specifically, if we need to create highly realistic and useful models and simulations, we need precise parameterization. And the Bayesian method has made this possible in a much larger number of cases. According to some authors, the application of the Bayesian inference method (Bayesian statistics) marked the epidemiology of the early 21st century. The particular advantage of the Bayesian approach important in epidemiology is in solving problems where the number of parameters is higher than the number of degrees of freedom. In such situations, the estimation of the parameters by frequentist methods is extremely difficult or impossible. Joseph et al (8), as early as in 1995, showed that Bayesian method could be used for the evaluation of new diagnostic tests in the absence of a gold standard (which is almost never achieved because 100% sensitivity and 100% specificity can hardly be achieved in practice) and with a relatively few samples. In the last three decades, software solutions to the application of Bayesian methods have evolved so much that they have become an integral part of all serious statistical software programs.

In addition to predicting the number of infected persons, contemporary epidemiology also deals with the spatial scope of epidemics and their spread in time and space. Spatiotemporal epidemiological simulations are created by autocorrelation models. To obtain common multivariate distributions of random vectors, which is necessary for the spatial modeling, the so-called conditional autoregressive models are used, and Bayesian method is increasingly employed to evaluate the parameters of these models. In addition, the most significant advantages (and reasons for the continued rise in popularity) of the Bayesian approach are, on the one hand, its high flexibility and adaptability to testing and research or measurement conditions, and on the other, the typical application of the MCMC (Markov Chain Monte Carlo) test method, which allows the testing of a wide range of different statistical models, ie, distributions.

Outstanding examples of the application of Bayesian statistics in the epidemiological study of measles were given by Cherian et al (9), who investigated the spatiotemporal dynamics of two measles virus genotypes to control the spread of measles in the Indian subcontinent. Similarly, Del Fava et al (10) used Bayesian mixture modeling of serological data to estimate the seroprevalence of measles by age and to distinguish between groups of individuals with different degrees of miscommunication.

Further development of hardware and software, machine and deep learning and artificial intelligence, along with the development of epidemiological auto-adaptive models and simulations, will very soon bring new hyper-realistic simulations that will significantly contribute to the eradication of a vast majority of infectious diseases. The exponential growth of the human population, along with a range of sociological phenomena such as anti-vaxers, does not, of course, help combat infections. However, with the education of population in general and improving the IT literacy of health care staff, and investment in the development of modern epidemiological research methods, we can significantly increase our chances in this fight.
